# Evidence that natural selection maintains genetic variation for sleep in *Drosophila melanogaster*

**DOI:** 10.1186/s12862-015-0316-2

**Published:** 2015-03-13

**Authors:** Nicolas Svetec, Li Zhao, Perot Saelao, Joanna C Chiu, David J Begun

**Affiliations:** Department of Evolution and Ecology, University of California, 3352 Storer Hall, One Shields Ave., Davis, CA 95618 USA; Department of Entomology and Nematology, University of California, Davis, CA USA

**Keywords:** *Drosophila melanogaster*, Latitudinal cline, Spatially varying selection, Locomotor activity, Sleep, Circadian rhythms, Gene expression, RNA-seq

## Abstract

**Background:**

*Drosophila melanogaster* often shows correlations between latitude and phenotypic or genetic variation on different continents, which suggests local adaptation with respect to a heterogeneous environment. Previous phenotypic analyses of latitudinal clines have investigated mainly physiological, morphological, or life-history traits. Here, we studied latitudinal variation in sleep in *D. melanogaster* populations from North and Central America. In parallel, we used RNA-seq to identify interpopulation gene expression differences.

**Results:**

We found that in *D. melanogaster* the average nighttime sleep bout duration exhibits a latitudinal cline such that sleep bouts of equatorial populations are roughly twice as long as those of temperate populations. Interestingly, this pattern of latitudinal variation is not observed for any daytime measure of activity or sleep. We also found evidence for geographic variation for sunrise anticipation. Our RNA-seq experiment carried out on heads from a low and high latitude population identified a large number of gene expression differences, most of which were time dependent. Differentially expressed genes were enriched in circadian regulated genes and enriched in genes potentially under spatially varying selection.

**Conclusion:**

Our results are consistent with a mechanistic and selective decoupling of nighttime and daytime activity. Furthermore, the present study suggests that natural selection plays a major role in generating transcriptomic variation associated with circadian behaviors. Finally, we identified genomic variants plausibly causally associated with the observed behavioral and transcriptomic variation.

**Electronic supplementary material:**

The online version of this article (doi:10.1186/s12862-015-0316-2) contains supplementary material, which is available to authorized users.

## Background

Understanding how local adaptation maintains phenotypic and genetic differentiation in spite of high rates of gene flow is an important question in evolutionary biology [1]. Latitudinal clines have been of particular interest because many organisms and traits show patterns of geographic variation consistent with locally varying selective forces correlated with latitude [2]. In the model species *Drosophila melanogaster*, numerous phenotypic traits (reviewed in [3]), including some related to circadian behaviors [4] are correlated with latitude suggesting they are shaped by spatially varying selection.

However, despite their connection to circadian and locomotor activity rhythms, the population processes maintaining genetic variation for sleep have never been investigated. Sleep in *Drosophila* has been defined as 5 or more consecutive minutes of inactivity [5,6]. This definition has been subsequently validated electrophysiologically [7,8]. *Drosophila* sleep resembles mammalian sleep in many aspects. For example, both are characterized by an increased arousal threshold, the adoption of a particular posture, and for both, sleep bout duration varies with age and sex [5,6,9]. *Drosophila* sleep is, in addition, sensitive to the same pharmacological agents as mammalian sleep, and is constituted of different sleep phases that are determined by circadian and homeostatic mechanisms [5,6,8]. Finally, sleep deprivation impairs fly cognitive abilities [10-12] and, in cases of long-term deprivation, can lead to death [13]. For all these reasons, *D. melanogaster* has become an important model species for identifying the mechanisms underlying the regulation of sleep [14,15], with the ultimate goal of improving our understanding of human sleep disorders [16].

The work presented here reports the first analysis of natural geographic variation in sleep in *D. melanogaster*. We found that flies from higher latitudes sleep substantially less than those from lower latitudes. High latitude flies also show a phase shift consistent with greater sunrise anticipation than that of lower latitude flies. Our analysis of the head transcriptome for the two populations showing the greatest sleep differences revealed that most gene expression differences between populations are circadian-time dependent and provided potentially valuable molecular insights into the observed behavioral phenotypes.

## Results and discussion

**Figure 1:**
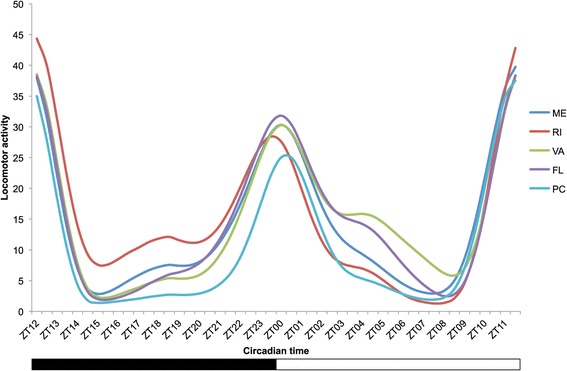
**Geographic variation in locomotor activity among five American** ***D. melanogaster*** **populations.** The graph was obtained from the Eduction analysis of FaasX software. The white bar underneath the graph represents the photophase, the black bar the scotophase. The populations are Maine (ME; latitude: 44°37′N), Rhode Island (RI; 41°49′N), Virginia (VA; 37°32′N), Florida (FL, 30°20′N) and Panama City (PC; 8°58′N). The raw locomotor activity profiles are shown on Additional file 1: Figure S2.

**Figure 2:**
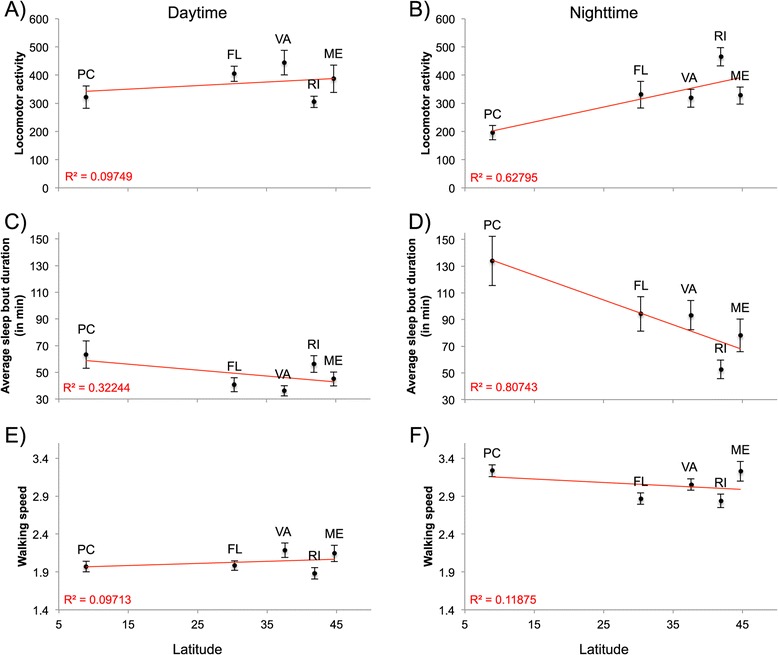
**Latitudinal cline in nighttime sleep bout duration.** Daytime and nighttime population mean phenotypic values (± s.e.m.) are shown respectively for locomotor activity **A)** and **B)**, sleep bout duration **C)** and **D)**, and walking speed **E)** and **F)**. Only sleep bout duration follows a latitudinal cline at nighttime (linear regression: R-square = 0.80; p-value = 0.038) but not at daytime (linear regression: R-square = 0.31; p = 0.32). The red line represents the regression of population phenotypic means (y-axis) over latitude (x-axis). ME: Maine, RI: Rhode Island, VA: Virginia, FL: Florida, and PC: Panama City.

**Figure 3:**
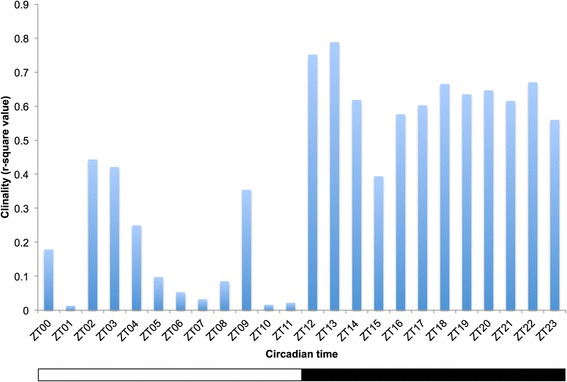
**Correlations with latitude for sleep phenotype measured at different circadian times.** The histogram bars correspond to the R-square values obtained when the hour-by-hour average sleep bout duration was regressed over population latitude. The fact that none of the 24 regressions was significant after correction for multiple testing but nighttime regressions showing elevated R-square is consistent with the presence of a global daytime *vs.* nighttime sleep pattern rather than sharp differences occurring over short time periods. The white bar underneath the graph represents the photophase, the black bar the scotophase.

*D. melanogaster* males sampled from populations collected along a latitudinal gradient ranging from Maine (USA; 44°N) to Panama City (Panama; 8°N) were entrained under semi-natural conditions (*i.e.* oscillating light and temperature; for more details see Methods and Additional file 1: Figure S1) prior to measurement of their locomotor activity. Nighttime locomotor activity profiles from higher and the lower latitudes differ substantially (Figure 1, Additional file 1: Figure S2). The regressions over latitude of the average locomotor activity during the photophase and the scotophase (Figure 2A and B respectively) show that nighttime locomotor activity is more strongly correlated with latitude (R-square = 0.62) than daytime locomotor activity (R-square = 0.09) suggesting a contrast between nighttime *vs.* daytime patterns. To further investigate population differences, locomotor activity was parsed into two main components: sleep (*i.e.* average sleep bout duration; Figure 2C and D) and walking speed (Figure 2E and F). The former showed a very strong relationship with latitude, which explained 80% of the observed phenotypic variation (p = 0.03; Figure 2D); the difference in average sleep bout duration between temperate and equatorial populations was about two-fold. Both sleep duration and sleep bout number contribute to the observed pattern (see Additional file 1: Figure S3). The observation that nighttime walking speed shows no evidence of latitudinal variation (Figure 2E and F) supports the idea that sleep (*i.e.* the bouts of inactivity), rather than walking speed (*i.e.* the absolute number of infrared beam crosses), constitutes the key behavioral difference in nighttime activity levels in higher *vs.* lower latitude populations. To our knowledge this is the first demonstration of genetically determined geographic differentiation for sleep behavior in *Drosophila*. Importantly, the different patterns of geographic variation of sleep do not result from sharp differences occurring over short time periods (Figure 3), but rather, from a general night versus day pattern. These observations support the idea that nighttime and daytime sleep are mechanistically distinct [17-20] and may evolve independently.

**Figure 4:**
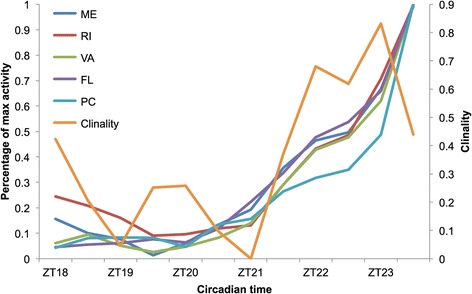
**Geographic variation in sunrise anticipation.** Percentage of maximal activity across time (= late night activity level) was a normalization of each line’s activity profile between 0 and 1. The data were then averaged over lines for each population. R-squares were obtained at each circadian timepoint from the regression of population mean activity levels over latitude.

Population average locomotor activity patterns (Figure 1) suggest that the few hours preceding the morning light transition also exhibit geographic variation. In particular, the phase of the morning peak (around ZT00) of the Rhode Island (RI) population occurs before light transition whereas it occurs after light transition for Panama City (PC), (with Maine (ME), Virginia (VA) and Florida (FL) being intermediate), which would be consistent with genetic variation in the anticipation of sunrise. To investigate this hypothesis, we quantified ramping in activity relative to the nighttime maximum locomotor activity (Figure 4 and see material and methods normalization details). This analysis revealed substantial geographic variation for ramping activity. For example, at ZT23 the PC population had reached only 50% of maximal activity, whereas flies from northern latitudes were already at 75% of their maximum activity; latitude explains 83% of the variation in late night activity level at this timepoint (regression; p-value = 0.026).

To investigate this further, we carried out an experiment in which a set of PC and ME flies were entrained in LD (Light/Dark) with fluctuating light and temperature using the same incubator program as in the aforementioned experiment. After 3 days of entrainment the flies were shifted to constant dark (DD) conditions with constant temperature (mean temperature of the LD phase). Examination of the activity profiles under DD conditions revealed the persistence of the morning activity peak. Moreover, the phase of this peak in ME flies was about 1 hour earlier than the PC peak (ME phase = 6.1 ± 0.44; PC phase = 7.2 ± 0.28; W/KW test: p = 0.02) but the free running periods were unchanged (period_PC_ = 24.7; period_ME_ = 24.6; W/KW test: p = 0.3). This is consistent with a genetically determined difference in sunrise anticipation between high and low latitude flies. This result contrasts with previous work studying alternative clock mutants under naturalistic conditions [21]. However, the two studies differ in important ways, including entrainment regimes and experimental genotypes. Moreover, we cannot rule out an alternative hypothesis that differential anticipation is driven by population differences in thermal sensitivity rather than pure circadian variation *per se*. We found no evidence of evening peak phase differences between PC and ME (ME phase = 17.4 ± 0.4; PC phase = 16.5 ± 0.3; W/KW test: p = 0.08). This phenomenon is reminiscent of splicing efficiency of *dmpi8* intron of *period* [22], where evening peaks are shifted by temperature without changes in period length. However, we found no evidence of variation in *dmpi8* splicing efficiency across latitude (see Additional file 2).

To investigate potential molecular underpinnings of behavioral differences between high and low latitude populations, we carried out a transcriptomic analysis of male heads sampled at different circadian times from Panama City (PC) and Rhode Island (RI) populations (Methods). We chose these two populations because they exhibited the greatest difference in sleep (Figure 1). Of the 13072 genes expressed in our data, 2119 (16%) showed expression differences (FDR = 0.05) between populations for at least one timepoint (see Additional file 3 for full list of the differentially expressed genes). However, only 7% of the 2119 genes were differentially expressed at all four timepoints. In other words, 93% of the geographic differences in gene expression were timepoint dependent, which strongly suggests that a comprehensive description of geographic differentiation in *D. melanogaster* transcript abundance may require sampling that accounts for circadian time.

Genes that in previously published experiments exhibited cycling polyA mRNA abundance in heads [23], brain cycling expression [24], entrainment by light or temperature [25], or direct regulation by the key circadian transcription activator CLOCK [26] showed significant enrichments among the genes exhibiting geographic differentiation in expression in our data (see Additional file 2 for more details). This suggests that a significant component of the geographic variation in mRNA abundance in the fly head is influenced by circadian regulation. We found no support for the idea that polymorphic chromosome inversions play an important role in geographic variation in gene expression (see Additional file 2).

**Figure 5:**
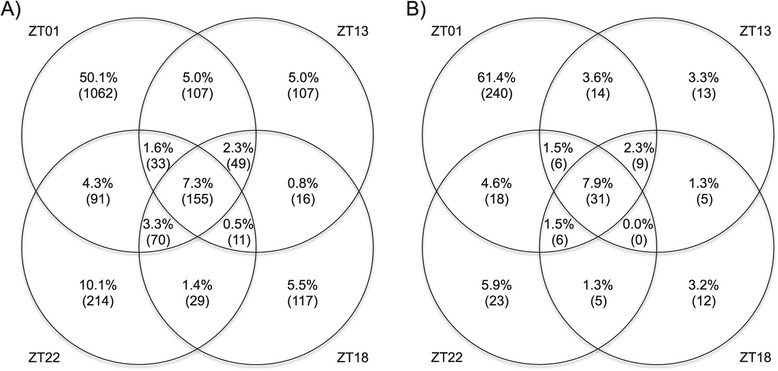
**A large fraction of the geographic variation in gene expression is circadian time specific. A)** Venn Diagram showing the distribution across timepoints of the genes differentially expressed between PC and RI. **B)** Venn Diagram showing the distribution across timepoints of the genes differentially expressed and located into the 5% outlier F_ST_ windows. Actual number of genes is indicated in parenthesis.

The timepoint at which the greatest number of genes were differentially expressed was ZT01 (Figure 5A), at which 76% (N = 1612) of the genes showed geographic variation in expression, as compared to ZT13, ZT18 and ZT22, where respectively 23% (N = 491), 23% (N = 492), and 29% (N = 616) of the expressed genes showed geographic differences. In addition, 66% of the genes differentially expressed at ZT01 were not differentially expressed at other timepoints. The ZT01 timepoint is unique in several respects: (1) it is our only daytime timepoint, (2) it is a peak activity time, and (3) it corresponds to a transition time between night and day. Moreover, the fact our experimental lights turned on suddenly in the morning rather than ramping smoothly could have contributed to the strong expression differences observed at ZT01.

**Figure 6:**
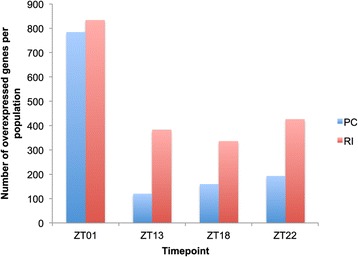
**Variation in the direction of the expression difference.** The figure shows the number of genes significantly overexpressed in PC *vs.* RI (blue) and RI *vs.* PC (red) for each timepoint.

At ZT01 there is no obvious pattern of greater transcript abundance being more common in one population or the other (Figure 6), though the magnitude of expression difference between populations is greater for genes expressed at a higher level in RI compared to genes expressed at a higher level in PC (average fold change for RI = 2.47; for PC = 1.42; MW test: p < 0.0001). The remaining timepoints, all of which are nighttime, show a different pattern (Figure 6), with a greater proportion of differentially expressed genes showing higher mRNA abundance in RI than in PC. Thus, it appears that population differences in transcriptional activity show some degree of correlation with population differences in nighttime activity. This is consistent with previous gene expression analyses showing that transcription levels in *Drosophila* heads are higher in awake *vs.* sleeping individuals [27].

To investigate the possible role of natural selection in generating the observed population differences in gene expression we characterized the association between differentially expressed genes and population genetic differentiation as summarized by *F*_*ST*_. If cis-acting regulatory variants [28] influenced by differential selection in high *vs.* low latitude populations play an important role in gene expression differences, then gene regions near differentially expressed genes may be associated with unusually high levels of genetic differentiation relative to genome wide averages [29,30]. We used our gene expression data and published population genomic data from Maine and Florida *D. melanogaster* populations [31] to test this hypothesis. We identified the differentially expressed genes that overlapped the 1-kb windows associated with the 5% most extreme average *F*_*ST*_ in Reinhardt *et al.* [31] which are likely enriched with targets of spatially varying selection. Out of the 1854 expressed genes that fell within an outlier *F*_*ST*_ window, 391 (21%) showed expression differences for at least one time-point (Figure 5B). Thus, outlier *F*_*ST*_ windows are significantly enriched in differentially expressed genes compared to the genome average (16%, 2119 differentially expressed genes out of a total of 13072 genes; hypergeometric test: p < 1.4 × 10^−9^). Expressed genes that are located within the outlier *F*_*ST*_ windows are 30% more likely to show expression difference between populations than genes not located in such windows. ZT01 contributed substantially to this enrichment, as 17.8% (330) of ZT01 differentially expressed genes were located in the outlier *F*_*ST*_ windows (out of 1854) and only 12.3% (1612 out of 13072) were differentially expressed (Figure 5B). For the three other timepoints, the outlier *F*_*ST*_ windows contain an excess of 5 to 16% of differentially expressed genes as compared to the genome average, but the enrichments are not statistically significant, perhaps due to reduced statistical power. Overall, these findings support the idea that the observed expression differences between populations are significantly influenced by *cis*-regulatory variants associated with high levels of geographic differentiation.

To carry out a more directed analysis of differentially expressed genes that might be causally linked to the geographic variation in locomotor activity, we first identified the genes annotated with GO terms plausibly linked to activity rhythms (see Material and Methods); there were 242 such genes, of which 237 were expressed in our data. Seventy-six (32%) of these genes showed expression differences between populations (see Additional file 3 for the gene list), compared to the genome average of 16% (*i.e.* 2-fold enrichment; hypergeometric test: p = 8.8 × 10^−10^). Of these 76 genes, 21 overlapped a 5% outlier *F*_*ST*_ window, supporting the hypothesis that spatially varying selection plays an important role in shaping geographic differences in transcript abundance for genes related to locomotion/circadian rhythms.

In principle, the observed gene expression differences between PC and RI could arise due to genotypic differences or as plastic downstream consequences of behavioral differences (awake *vs.* sleeping). While population genomic data support the idea that the observed transcriptomic differences have a genetic basis tightly linked to the corresponding genes, we sought additional evidence bearing on this question. Cirelli *et al.* [27] identified 138 genes that showed expression differences for a single *D. melanogaster* genotype across the behavioral states, awake *vs.* sleeping. Of those, 133 were expressed in our data set (we refer below to them as BSE genes (Behavioral State dependent Expression). 55% (74) of BSE genes were differentially expressed between PC and RI (*vs.* a genome average of 16% of differentially expressed genes; hypergeometric test: p = 1.47 × 10^−25^), suggesting that some of the expression differences between PC and RI result from behavioral state differences. However, the BSE genes showing geographic variation in expression were significantly underrepresented in the 5% outlier *F*_*ST*_ windows (hypergeometric test: p = 0.02), and underrepresented in our list of 76 differentially expressed circadian rhythms candidates as well (hypergeometric test: p = 0.04). In other words, most of the genes exhibiting geographic differences that are also associated with circadian annotations are likely not influenced by behavioral state. Instead, they likely are causally related to behavioral state. Another possible explanation for population differences in expression is that in our experimental condition of relatively high temperature, one population, perhaps Rhode Island, is more thermally stressed. While we cannot rule out that such a phenomenon contributes to the observed differences, we observed no sign of enrichment for GO terms associated with heat, thermal response or stress in our gene expression analysis. This suggests that population differences in head gene expression as well as the locomotor activity/sleep patterns are unlikely to be explained in terms of differential stress responses.

The list of 76 differentially expressed genes (see Additional file 3) contains 6 genes that are either part of the core circadian clock or directly interact with the corresponding genes (*gl*, *Pdp1*, *Pdf*, *timeout*, *mnb*, and *nej*). In addition, *Atx-2* and *tyf*, both of which were differentially expressed at ZT01, form a protein complex that regulates *period* translation [32,33], suggesting that transcriptional and post-transcriptional regulation may contribute to geographic differences in locomotor activity. Two genes, *unc-80* and *na,* previously shown to be related to ramping in activity [34] were also differentially expressed between PC and RI. With the exception of *gl*, all the aforementioned genes are differentially expressed at ZT01. However, given that differences in ZT01 transcript abundance may not be manifest as downstream behavioral phenotypes for several hours, such differences may be less likely to play a primary role in nighttime sleep or sunrise anticipation.

Of the 76 candidate genes, only 24 are differentially expressed at nighttime (ZT13, ZT18, ZT22) and, among those, 10 that are differentially expressed at ZT13 and ZT18 seem more plausible candidates as contributors to variation in nighttime sleep or sunrise anticipation (*Ddc*, *Hsp83*, *robo*, *Rh5*, *Acer*, *Dat*, *Rh6*, *slmo*, *Zip42C.1*, *to*, *Mhc*, and *Irk1*). *Ddc* and *Dat*, which were differentially expressed at ZT13, are part of the dopamine/serotonin pathway. *Ddc* is located in an outlier *F*_*ST*_ window from Reinhardt *et al.* [31]. The DDC protein catalyzes the last step in the production of dopamine and serotonin [35], both of which are important neurotransmitters for the regulation of the sleep/wake cycle [36]. Another important gene for sleep homeostasis [13], *Hsp83*, showed circadian cycling behavior in Rodriguez *et al.* [23], was overexpressed in PC at both ZT13 and ZT22, and was spanned by an outlier *F*_*ST*_ window. Finally, two other genes in this candidate list, *Acer* and *takeout*, both of which were differentially expressed at all timepoints, are worth mentioning. An *Acer* null allele affects nighttime sleep [37] and is one of the few sleep-annotated genes showing geographic variation in expression at nighttime. *takeout (to)* shows interesting patterns at several levels. It is significantly overexpressed in RI at all 4 timepoints with one of the greatest expression fold change differences between populations (2.8 fold change on average across timepoints). Both nascent transcripts and polyA mRNA transcripts of *takeout* cycle throughout the day [23]. Additionally, *takeout* is entrained by light and temperature [25]. It appears to be located immediately downstream from the core clock pathway, as its expression is regulated by *Clock* [26] and *Pdp1* [38] - the latter also showing evidence of geographic variation in expression at ZT01 in our study. Finally, the *takeout* locus is located in a genomic region showing strong latitudinal differentiation [31,39].

## Conclusion

While the genetics of sleep have been studied in several model systems, the population processes maintaining genetic variation for sleep have received little attention. Here we suggest that there is a selectively maintained latitudinal cline in nighttime sleep, as well as evidence of geographic variation for sunrise anticipation. These data, along with our gene expression and existing population genomic data, support the idea that a significant component of this locomotor and gene expression variation results from selection in heterogeneous environments, though the mechanistic connection between variation in sleep and fitness variation remains a mystery. Our data suggest that various biological processes may influence sleep variation, including dopamine/serotonin metabolism and post-transcriptional regulation of core clock components.

In addition to detailed functional analysis of the genes and traits identified here (including proteomic analysis), several important basic questions remain. First, extending our analysis to additional population samples may solidify the evidence for clinality and provide better estimates of cline parameters. Second, given the evidence for sex-specific locomotor activity rhythms [40] and gene expression [41], further experiments to assess whether female sleep and head/brain gene expression patterns also show geographic variation may be interesting. Third, our investigation used a single set of temperature and light conditions. While our results provide very strong evidence of genetic differences in nighttime sleep between high and low latitude populations in this laboratory environment, we have no information regarding the expression of these genotypic differences in other light (i.e. photoperiod and types of transitions) or temperature environments, or the role of genotype × environment interaction in the maintenance of genetic variation for sleep in this species [25,42-44]. Finally, functional investigation of natural variation affecting sleep in the *D. melanogaster* model [14,45] may provide important insights into the mechanistic and population genetic explanation for genetic variation for sleep in other animals, including humans.

## Methods

### Fly lines

We used isofemale lines to study a total of five *D. melanogaster* population samples. Four originated from locations along the east coast of North America: ME in Fairfield, Maine (latitude: 44°37′N), RI in Providence, Rhode Island (41°49′N), VA in Richmond, Virginia (37°32′N), and FL in Jacksonville, Florida (30°20′N) (all sampled in September 2011). An additional population (PC) was sampled from Panama City, Panama (8°58′N) in January 2012. All *Drosophila* stocks were maintained independently at room temperature on a standard yeast-cornmeal-agar food medium.

### Experimental conditions

For each population, we phenotyped 8 randomly selected isofemale lines. For each isofemale line, we generated experimental animals by allowing groups of 10 to 15 parental flies to mate and lay eggs in a vial for 3–4 days. Those vials, which contain 4 ml standard food, were placed into an incubator at 25°C with 12:12 Light/Dark cycle and 50% humidity. Fly activity for experimental animals was measured following methods described in [46]. Young male offspring were collected within 12 hours of eclosion using light CO_2_ anesthesia. They were then aged for 3 days in groups of 5–7 until tested. Using light CO_2_, males were placed into activity tubes containing a nutritive medium (5% sucrose (Sigma, St. Louis, MO), 2% bacto agar (Difco, Sparks, MD) [46]) at one end and a foam plug at the other end. The activity tubes were then inserted into Trikinetics *Drosophila* Activity monitors (Trikinetics Inc., Waltham, MA). Finally, the monitors were placed into a Percival environmental chamber (Percival Scientific Inc., Perry, IA, USA) and locomotor activity data, based on infrared beam crosses in the middle of the activity tubes, were collected in 1-min. bins continuously for 9 days using DAMSystem software. Only the data from day 5 through 12 were used for analysis.

Light intensity and temperature fluctuated across the day (see Additional file 1: Figure S1). Temperature oscillated between a minimum of 25.6°C at ZT23 and a maximum of 29.4°C at ZT07, corresponding roughly to the temperatures on a tropical summer day. Lights were on a 12:12 light/dark cycle. Daily light fluctuations mimic days and nights, with higher midday light intensity (2 out of 2 banks of lights on between ZT04 and ZT08). A light bank consists in 2 Philips F20T12/CW fluorescent lamps, giving 20 W (corresponding to 1200 lumen) of white light each.

For the phase measures, a set of 64 flies (4 males from each of the 8 lines for both PC and ME) were entrained in the aforementioned environment for 3 days and were then switched to constant dark and constant temperature (27.5°C corresponding to the daily average temperature of the entrainment regime).

### Data processing and analyses

Locomotor activity data were processed as follows. The raw data were processed with Microsoft Excel 2011 macros (Microsoft, Redmond, WA, USA). Individuals that died during the course of the experiment, as well as those who exhibited any 24 consecutive hours with 24 or fewer infrared beam crosses were considered as non-informative and were discarded from the analysis. This represented a small number of individuals (21), which were randomly distributed among the lines (see Additional file 1: Table S1 for the sample sizes). We calculated locomotor activity as number of infrared beam cross per unit of time and walking speed as the number of infrared beam cross per unit of time excluding inactive time. We identified the sleep bouts by tracking any period of 5 (or more) minutes of inactivity in the raw activity data. We then measured sleep-related variables (average sleep duration, average sleep bout number and average sleep bout duration). As we did not detect any age effect component on the clinal pattern between the populations, all measures of activity and sleep were averaged across days for each individual. For the estimations of ramping in activity in Figure 4, the activity levels were normalized as follows: (A_t_-A_min_)/(A_max_-A_min_) where A_t_ is the number of infrared beam cross for a 30 min interval, and where A_min_ and A_max_ are respectively the minimum and the maximum activity of each line during the ZT18 to ZT23.5 using 30 minutes binned mean activity levels.

For each line, mean phenotypic variables were calculated by averaging the data of all individuals originating from the same line. All means and standard error of the mean (s.e.m.) were calculated by averaging the phenotypic means across lines sharing the same geographic origin. Phase and period estimations were performed with FaasX [47]. Statistical tests were performed with JMP software v10.0.0 (SAS institute Inc, Cary, NC, USA). As the data were not normally distributed even after transformation, we used non-parametric tests: Wilcoxon/Kruskal-Wallis tests (W/KW test), and performed Bonferroni corrections for multiple testing when appropriate.

### Sample preparation for RNA-seq

The sampling timepoints were chosen to correspond to those at which behavioral differences between populations were greatest. Given the results from our phenotypic analysis, we chose 4 timepoints at which behavioral clinality was high with a bias toward nighttime (ZT13, ZT18 and ZT22), plus a morning daytime timepoint ZT01. For each of the two populations that showed the strongest sleep differences (RI and PC), we generated experimental animals as described for the phenotypic experiment. Flies were reared in a similar manner as described for the phenotypic experiment except they were aged and then entrained into individual standard vials with 4 ml food. After four days of entrainment (7 days-old), four flies from each isofemale line were flash frozen in liquid nitrogen at timepoints ZT01, ZT13, ZT18 and ZT22. We combined flies from each of the 25 lines from either the RI population and that from PC population. One biological replicate consisted of a pool of 50 individuals (2 males from each of 25 isofemale lines, among which were the same 8 lines used in the behavioral experiments). Flies collected at night were flash frozen under red light. Following snap freezing, fly heads were collected and immediately transferred to Trizol for RNA extraction. Poly(A) + RNA was prepared using an NEB mRNA isolation module (E7490S). RNA-seq libraries were constructed using NEB kits E7530S (library prep), and E7335S (Oligos). Libraries were constructed following the manufacturer instructions with only one exception; we used Aline Bioscence PCR CleanDX beads for the DNA purification steps. Individual libraries were constructed with insert size between 180–200 bp and sequenced by BGI Americas (Cambridge, MA, USA) on an Illumina Hiseq2000 platform using paired-ends chemistry and 100 cycles.

### Data analysis

In total, we generated 587 million cleaned paired-end reads for 16 libraries (*i.e.* an average of 36 million reads per library; see Additional file 1: Table S2). Clean reads were deposited to NCBI. Filtered clean reads (Q > 20 for amino acid and Q > 30 for read) in each sample or replicate were aligned independently to the *D. melanogaster* reference genome (FlyBase r5.55) using Bowtie-based TopHat [48] program. Our experiment showed high degree of replication, with R-squares > 0.99 for all 8 pairs of biological replicates (see Additional file 1: Figure S4). We adopted Bedtools [49] to estimate read count of each gene, and then measured the differential expression using the Bioconductor package (version 2.14) in R, including DESeq2 (version 1.4.5), edgeR (version 3.0.8) and voom-limma (version 3.20.8). The Benjamini–Hochberg procedure was used to control the false discovery rate [50] for all methods. Genes with a minimum of 10 counts in average across the 16 libraries were kept for further analysis. Here, we present results from DESeq2 differentially expressed genes because these results showed the greatest consistency with the other two methods. We also measured isoform expression changes by Cufflinks [51]. For the above differential expression and analysis, both Flybase r5.55 annotation and modENCODE annotation [41] were used. We compared differential gene expression at each timepoint (ZT01, ZT13, ZT18 and ZT22) between Panama City and Rhode Island populations.

### Functional annotation and enrichments

Candidate sleep genes were defined as those associated with GO terms linked to activity rhythms (all GOs containing the following keywords: sleep, catecholamine, dopamine, serotonine, circadian rhythms, and locomotion behavior). Enrichments of differentially expressed genes in alternative gene classes or locations were estimated by hypergeometric tests.

### Availability of supporting data

The data sets supporting the results of this article are available in the SRA archive from NCBI repository under the accession number SRP052570. http://www.ncbi.nlm.nih.gov/Traces/sra/?study=SRP052570.

## Additional files

Additional file 1:
**Supplementary online figures.**
Additional file 2:
**Supplementary online results and discussion.**
Additional file 3:
**RNAseq result table.**

## Abbreviations

ME: Maine
RI: Rhode Island
VA: Virginia
FL: Florida
PC: Panama City
*F*_*ST*_: Fixation index
GO: Gene ontology
LD: Light/Dark
DD: Dark/Dark
ZT: Zeitgeber
BSE: Behavioral State dependent expression
