# Supplementary material for: Evidence that natural selection maintains genetic variation for sleep in Drosophila melanogaster
Source: BMC Evol Biol. 2015 Mar 13;15:41. doi: 10.1186/s12862-015-0316-2 (PMC4374177; doi:10.1186/s12862-015-0316-2)
Supplement: Additional file 1: — Supplementary online figures. [file 12862_2015_316_MOESM1_ESM.docx]

Supplementary Online Figure S1: Daily temperature and light fluctuations during entrainment.

Supplementary Online Figure S2: Locomotor activity pattern based on raw data. The figure represents the average number of red beam cross (activity count) per 30 min per fly across the 7 days of the experiment. The white bar underneath the graph represents the photophase, the black bar the scotophase.

Supplementary Online Figure S3: Latitudinal variation in sleep duration and sleep bout number in American populations of *D. melanogaster*. Panels A) and B) respectively show the average (± s.e.m.) daytime and nighttime sleep duration of each population sample. C) and D) correspond to the average (± s.e.m.) daytime and nighttime sleep number. Sample sizes for each population are between N=56 and N=109 (see Supplementary online Table 1 for more details). In each panel the linear regression of the y-axis parameter against latitude and the corresponding R-square value are shown.

Supplementary Online Figure S4: Biological replication in the RNA-seq experiment. Each panel represents the correlation of gene expression measured between biological replicates for each population and timepoint combination.

Supplementary online Table 1: Sample sizes for each line that was phenotyped.

| **Line** | **N** |  | **Line** | **N** |  | **Line** | **N** |  | **Line** | **N** |  | **Line** | **N** |
| --- | --- | --- | --- | --- | --- | --- | --- | --- | --- | --- | --- | --- | --- |
| PC1 | 10 |  | FL1 | 8 |  | VA1 | 8 |  | RI1 | 7 |  | ME1 | 12 |
| PC2 | 18 |  | FL2 | 8 |  | VA2 | 8 |  | RI2 | 8 |  | ME2 | 9 |
| PC3 | 19 |  | FL3 | 8 |  | VA3 | 7 |  | RI3 | 8 |  | ME3 | 14 |
| PC4 | 12 |  | FL4 | 8 |  | VA4 | 7 |  | RI4 | 8 |  | ME4 | 12 |
| PC5 | 16 |  | FL5 | 5 |  | VA5 | 8 |  | RI5 | 8 |  | ME5 | 11 |
| PC6 | 15 |  | FL6 | 9 |  | VA6 | 8 |  | RI6 | 7 |  | ME6 | 12 |
| PC7 | 14 |  | FL7 | 7 |  | VA7 | 8 |  | RI7 | 4 |  | ME7 | 12 |
| PC8 | 5 |  | FL8 | 7 |  | VA8 | 7 |  | RI8 | 7 |  | ME8 | 12 |
| **PC_tot_** | **109** |  | **FL_tot_** | **56** |  | **VA_tot_** | **61** |  | **RI_tot_** | **57** |  | **ME_tot_** | **94** |

Supplementary online Table 2: read numbers obtained for each RNA-seq library.

| **Population** | **Timepoint** | **Replicate** | **Reads number (million)** | **Total data (million BP)** |
| --- | --- | --- | --- | --- |
| Panama | ZT01 | Replicate1 | 38.56 | 3856.42 |
| Panama | ZT01 | Replicate2 | 33.99 | 3398.52 |
| Panama | ZT13 | Replicate1 | 33.51 | 3351.21 |
| Panama | ZT13 | Replicate2 | 39.07 | 3907.19 |
| Panama | ZT18 | Replicate1 | 33.41 | 3340.67 |
| Panama | ZT18 | Replicate2 | 33.53 | 3353.13 |
| Panama | ZT22 | Replicate1 | 36.14 | 3614.47 |
| Panama | ZT22 | Replicate2 | 42.98 | 4298.11 |
| Rhode island | ZT01 | Replicate1 | 36.77 | 3676.86 |
| Rhode island | ZT01 | Replicate2 | 37.86 | 3786.41 |
| Rhode island | ZT13 | Replicate1 | 37.04 | 3704.20 |
| Rhode island | ZT13 | Replicate2 | 41.46 | 4145.97 |
| Rhode island | ZT18 | Replicate1 | 31.78 | 3177.63 |
| Rhode island | ZT18 | Replicate2 | 32.29 | 3228.63 |
| Rhode island | ZT22 | Replicate1 | 39.98 | 3998.50 |
| Rhode island | ZT22 | Replicate2 | 38.73 | 3873.42 |
| Total |  |  | 587.11 | 58711.33 |
