# Supplementary material for: Evidence that natural selection maintains genetic variation for sleep in Drosophila melanogaster
Source: BMC Evol Biol. 2015 Mar 13;15:41. doi: 10.1186/s12862-015-0316-2 (PMC4374177; doi:10.1186/s12862-015-0316-2)
Supplement: Additional file 2: — Supplementary online results and discussion. [file 12862_2015_316_MOESM2_ESM.docx]

**Evidence that natural selection maintains genetic variation for sleep in *Drosophila melanogaster***

Nicolas Svetec*^1^, Li Zhao^1^, Perot Saelao^1^, Joanna C. Chiu^2^, and David J. Begun^1^

**Supplementary Results and Discussion**

**Distribution of the differentially expressed gene across chromosomes and inversions.**

We looked for chromosome enrichments for genes showing significant geographic expression differences.

*Table 1: p-values of hypergeometric tests for enrichment in differentially expressed genes across chromosome arms. Green cells indicate significant p-values after Bonferroni correction for multiple testing*.

| Chromosome Arm | X | 2L | 2R | 3L | 3R | 4 |
| --- | --- | --- | --- | --- | --- | --- |
| ZT01 | 0.059 | 0.885 | 0.266 | 0.929 | 0.353 | 0.524 |
| ZT13 | 0.993 | 0.978 | 0.143 | 0.678 | 0.000 | 1.000 |
| ZT18 | 0.975 | 0.952 | 0.005 | 0.643 | 0.263 | 0.354 |
| ZT22 | 0.881 | 0.978 | 0.875 | 0.168 | 0.003 | 0.546 |
| All ZTs | 0.976 | 0.985 | 0.489 | 0.205 | 0.007 | 1.000 |
| At least one timepoint | 0.130 | 0.961 | 0.277 | 0.950 | 0.062 | 0.632 |

For each of 6 categories (genes differentially expressed at: ZT01, ZT13, ZT18, ZT22, all ZTs, and at least one timepoint), we tested whether any chromosome arms were enriched for differentially expressed genes (Table 1). We found significant enrichments for chromosome 3R at ZT13, ZT22, and for genes differentially expressed at all timepoints. These three enrichments might be linked, as the genes expressed at all timepoints contribute to 28% and 30% of the genes differentially expressed at ZT13 and ZT18, respectively. We also found significant enrichment on chromosome 2R at ZT18. As in *D. melanogaster* the frequency of some chromosomal inversions are known to vary clinally, we determined whether those chromosome arm enrichments could be related to common inversions.

*Table 2: p-values of hypergeometric tests for enrichment in differentially expressed genes across chromosome inversions. There were no significant p-values after Bonferroni correction for multiple testing.*

| Inversion | *(3R)P* | *(3R)Mo* | *(2R)Ns* | *(3R)K* | *(3L)P* | *(2L)t* |
| --- | --- | --- | --- | --- | --- | --- |
| At least one timepoint | 0.710 | 0.589 | 0.207 | 0.290 | 0.185 | 0.942 |
| ZT01 | 0.723 | 0.718 | 0.523 | 0.349 | 0.257 | 0.745 |
| ZT13 | 0.330 | 0.676 | 0.043 | 0.025 | 0.048 | 0.823 |
| ZT18 | 0.616 | 0.609 | 0.012 | 0.586 | 0.066 | 0.733 |
| ZT22 | 0.389 | 0.056 | 0.705 | 0.060 | 0.037 | 0.711 |
| All ZTs | 0.122 | 0.275 | 0.717 | 0.019 | 0.044 | 0.430 |

We compared the proportion of differentially expressed genes in regions spanned by *In(3R)P*, *In(3R)Mo*, *In(3L)P*, *In(2L)t*, *In(2R)Ns*, and *In(3R)K* relative to autosomal regions not spanned by inversions. Though there is a slight enrichment of differentially expressed genes in *In(2R)Ns*, *In(3R)K* and *In(3L)P* (Table 2), none of the regions spanned by these inversions were significant after Bonferroni correction. While this does not rule out an influence of inversions on geographic expression differences, it suggests that at best their role is minor.

**Enrichment in genes linked to circadian functions**

*Table 3: Enrichment in circadian regulated genes in the differentially expressed (DE) genes between Rhode Island (RI) and Panama City (PC).*

| Data set | Study | %of DE genes in the corresponding data set | % of DE genes between PC and RI | Fold enrichment | p-value |
| --- | --- | --- | --- | --- | --- |
| Gene with cycling poly(A) mRNA expression | Rodriguez *et al*. [1] | 30 | 16 | 1.88 | 9×10^-13^ |
| Genes with at least one transcript showing cycling mRNA expression | Hughes *et al*. [2] | 23 | 16 | 1.44 | 2×10^-4^ |
| Genes entrained by light | Boothroyd *et al*. [3] | 37 | 16 | 2.31 | 2.1×10^-11^ |
| Genes entrained by temperature | Boothroyd *et al*. [3] | 28 | 16 | 1.75 | 2.6×10^-9^ |
| Genes regulated by CLOCK | Abruzzi *et al*. [4] | 31 | 16 | 1.94 | 1.2×10^-15^ |
| Genes differentially expressed between behavioral states (awake *vs*. sleep) | Cirelli *et al*. [5] | 55 | 16 | 3.44 | 1.5×10^-25^ |

Given that our populations showed differences in sleep, we asked how many of our differentially expressed genes were involved in circadian functions. Genes differentially expressed between RI and PC contained a 2-fold enrichment (30% of the cycling genes are differentially expressed *vs.* a genome average of 16%; hypergeometric test p= 9×10^-13^ ) of genes with cycling poly(A) RNA expression [1]. As our experimental animals experienced fluctuations in both light and temperature, we asked whether differentially expressed genes overlapped with genes entrained by light or genes entrained by temperature by comparing our list of differentially expressed genes with those reported in Boothroyd *et al*. [3]. We found that 37% of the genes entrained by temperature and 38% of the genes entrained by light were significantly differentially expressed between populations (about 2-fold enrichment as compared to the genome average: 16%; hypergeometric test: p = 2.6×10^-9^ and p = 2.1×10^-11^ respectively). However, as both light and temperature lists show large overlap, there is little power to detect whether light or temperature is the major entrainment factor.

To investigate the possible contribution of the core circadian clock in temporal dependent geographic differences in the head transcriptome, we compared our differentially expressed genes to the list of genes that may be transcriptionally regulated by CLOCK [4]. Of the 473 genes expressed in both studies, 145 (31%) showed geographic variation in their expression, while only 16% of all expressed genes showed geographic variation in their expression. This constitutes a nearly 2-fold enrichment (hypergeometric test: p = 1.15×10^-15^). In addition, a large majority of geographically differentially expressed genes (126 out of 145) were differentially expressed at ZT01, suggesting that at least a fraction of transcriptome differences between populations at this timepoint are directly driven by the core circadian clock output. All together, these results support the hypothesis that the differentially expressed genes are enriched in those regulating or being regulated by a circadian process.

***period* *dmpi8* splicing efficiency.**

The presence of a circadian regulated afternoon activity peak under natural/semi-natural conditions is subject to debate [6, 7]. We detected an increase in activity between ZT04 and ZT08 (when light intensity is the highest, Figure 1A) but it does not resemble the peak described in Vanin *et al*. [6]. Moreover, this increase in activity was higher for intermediate latitude populations (VA and FL) and lower for the cline end points (ME, RI and PC) providing no support for a cline in this activity component. However, in addition to likely genetic differences between the flies used here and in Vanin *et al*. [6], we did not used gradual light transitions and our temperatures were in a high thermal range, making it difficult to directly compare our results with those of Vanin *et al*. [6] and Varma *et al*. [7]

Recent studies documented the influence of the splicing of an intron (*dmpi8*) from the *period* gene on the midday sleep patterns [8–10]. We extracted RNA-seq reads spanning the intron and calculated the relative frequencies of the spliced and unspliced forms of *period* transcripts.

*Table 4: Splicing efficiency of the dmpi8 intron in PC and RI flies.*

| Timepoint | Population | # of reads with *dmpi8* intron | # of reads without *dmpi8* intron | Freq of unspliced *dmpi8* | PC *vs*. RI Fisher exact test p-value |
| --- | --- | --- | --- | --- | --- |
| ZT01 | PC | 12 | 45 | 0.21 | 0.14 |
|  | RI | 8 | 65 | 0.10 |  |
| ZT13 | PC | 56 | 318 | 0.14 | 0.62 |
|  | RI | 66 | 418 | 0.13 |  |
| ZT18 | PC | 53 | 255 | 0.17 | 0.50 |
|  | RI | 43 | 241 | 0.15 |  |
| ZT22 | PC | 18 | 73 | 0.19 | 0.31 |
|  | RI | 11 | 69 | 0.13 |  |

The largest difference between populations (roughly 2-fold) was at ZT01. However this difference was not significant (Fisher exact test PC *vs*. RI: p-value = 0.14).

The difference in intron splicing between populations might vary across the day, as we found stronger differences at ZT01 and ZT22 than ZT13 and ZT18. We examined our phenotypic data for a comparable signal to Cao *et al*. [10]. We found that sleep bout duration at ZT02 and ZT03 in particular followed a weak latitudinal trend (R-squares of about 0.4; Figure 2) but for neither timepoint was the regression of sleep bout duration *vs*. latitude for either timepoint was not found to be significant.

Supplementary References:

1. Rodriguez J, Tang C-HA, Khodor YL, Vodala S, Menet JS, Rosbash M. Nascent-Seq analysis of *Drosophila* cycling gene expression. Proc Natl Acad Sci U S A. 2013, 110:E275–84.

2. Hughes ME, Grant GR, Paquin C, Qian J, Nitabach MN. Deep sequencing the circadian and diurnal transcriptome of *Drosophila* brain. Genome Res. 2012, 22:1266–81.

3. Boothroyd CE, Wijnen H, Naef F, Saez L, Young MW. Integration of light and temperature in the regulation of circadian gene expression in *Drosophila*. PLoS Genet. 2007, 3:e54.

4. Abruzzi KC, Rodriguez J, Menet JS, Desrochers J, Zadina A, Luo W, Tkachev S, Rosbash M. *Drosophila* CLOCK target gene characterization: Implications for circadian tissue-specific gene expression. Genes Dev. 2011, 25:2374–2386.

5. Cirelli C, LaVaute TM, Tononi G: Sleep and wakefulness modulate gene expression in *Drosophila*. J Neurochem. 2005, 94:1411–9.

6. Vanin S, Bhutani S, Montelli S, Menegazzi P, Green EW, Pegoraro M, Sandrelli F, Costa R, Kyriacou CP. Unexpected features of *Drosophila* circadian behavioural rhythms under natural conditions. Nature. 2012, 484:371–5.

7. De J, Varma V, Saha S, Sheeba V, Sharma VK: Significance of activity peaks in fruit flies, *Drosophila melanogaster*, under seminatural conditions. Proc Natl Acad Sci U S A. 2013, 110:8984–9.

8. Majercak J, Sidote D, Hardin PE, Edery I. How a circadian clock adapts to seasonal decreases in temperature and day length. Neuron. 1999, 24:219–30.

9. Low KH, Chen W-F, Yildirim E, Edery I. Natural variation in the *Drosophila melanogaster* clock gene period modulates splicing of its 3’-terminal intron and mid-day siesta. PLoS One. 2012, 7:e49536.

10. Cao W, Edery I. A novel pathway for sensory-mediated arousal involves splicing of an intron in the *period* clock gene. SLEEP. 2015, 38:41–51.
